# Intranasal immunisation with recombinant *Toxoplasma gondii* uridine phosphorylase confers resistance against acute toxoplasmosis in mice[Fn FN1]

**DOI:** 10.1051/parasite/2023047

**Published:** 2023-11-02

**Authors:** Li-Tian Yin, Ying-Jie Ren, Yu-Jie You, Yong Yang, Zhi-Xin Wang, Hai-Long Wang

**Affiliations:** 1 Key Laboratory of Cellular Physiology, Ministry of Education, Department of Physiology, Shanxi Medical University Taiyuan 030001 Shanxi China; 2 School of Basic Medicine, Basic Medical Sciences Center, Shanxi Medical University Jinzhong Shanxi 030600 China

**Keywords:** *Toxoplasma gondii*, Uridine phosphorylase, Recombination protein, Intranasal immunisation, Mucosal vaccine

## Abstract

Toxoplasmosis is caused by *Toxoplasma gondii*, which infects all warm-blooded animals, including humans. Currently, control measures for *T. gondii* infection are insufficient due to the lack of effective medications or vaccines. In this paper, recombinant *T. gondii* uridine phosphorylase (r*Tg*UPase) was expressed in *Escherichia coli* and purified via Ni^2+^-NTA agarose. r*Tg*UPase was inoculated intranasally into BALB/c mice, and the induced immune responses were evaluated by mucosal and humoral antibody and cytokine assays and lymphoproliferative measurements. Moreover, the protective effect against the *T. gondii* RH strain infection was assessed by calculating the burdens of tachyzoites in the liver and brain and by recording the survival rate and time. Our results revealed that mice immunised with 30 μg r*Tg*UPase produced significantly higher levels of secretory IgA (sIgA) in nasal, intestinal, vaginal and vesical washes and synthesised higher levels of total IgG, IgG1 and, in particular, IgG2a in their blood sera. r*Tg*UPase immunisation increased the production of IFN-gamma, interleukin IL-2 and IL-4, but not IL-10 from isolated mouse spleen cells and enhanced splenocyte proliferation *in vitro*. r*Tg*UPase-inoculated mice were effectively protected against infection with the *T. gondii* RH strain, showing considerable reduction of tachyzoite burdens in liver and brain tissues after 30 days of infection, and a 44.29% increase in survival rate during an acute challenge. The above findings show that intranasal inoculation with r*Tg*UPase provoked mucosal, humoral and cellular immune responses and indicate that r*Tg*UPase might serve as a promising vaccine candidate for protecting against toxoplasmosis.

## Abstract (fr)

La toxoplasmose est causée par *Toxoplasma gondii*, qui infecte tous les animaux à sang chaud, y compris les humains. Actuellement, les mesures de contrôle de l’infection à *T. gondii* sont insuffisantes en raison du manque de médicaments ou de vaccins efficaces. Dans cet article, l’uridine phosphorylase recombinante de *T. gondii* (rTgUPase) a été exprimée dans *Escherichia coli* et purifiée via de l’agarose Ni2^+^-NTA. La rTgUPase a été inoculée par voie intranasale à des souris BALB/c et les réponses immunitaires induites ont été évaluées par des dosages d’anticorps et de cytokines muqueuses et humorales et par des mesures de lymphoprolifération. De plus, l’effet protecteur contre l’infection par la souche RH de *T. gondii* a été évalué en calculant la charge de tachyzoïtes dans le foie et le cerveau et en enregistrant le taux et la durée de survie. Nos résultats ont révélé que les souris immunisées avec 30 μg de rTgUPase produisaient des taux significativement plus élevés d’IgA sécrétoires (sIgA) dans les lavages nasaux, intestinaux, vaginaux et vésicaux et synthétisaient des taux plus élevés d’IgG totales, d’IgG1 et, en particulier, d’IgG2a dans leur sérum sanguin. L’immunisation par la rTgUPase a augmenté la production d’IFN-gamma, d’interleukine IL-2 et IL-4, mais pas d’IL-10 à partir de cellules de rate de souris isolées et a amélioré la prolifération des splénocytes *in vitro*. Les souris inoculées par la rTgUPase ont été efficacement protégées contre l’infection par la souche RH de *T. gondii*, montrant une réduction considérable de la charge de tachyzoïtes dans les tissus hépatiques et cérébraux après 30 jours d’infection et une augmentation de 44,29 % du taux de survie lors d’une épreuve aiguë. Les résultats ci-dessus montrent que l’inoculation intranasale de rTgUPase provoque des réponses immunitaires muqueuses, humorales et cellulaires et indiquent que la rTgUPase pourrait servir de candidat vaccin prometteur pour la protection contre la toxoplasmose.

## Introduction

Toxoplasmosis is a disease caused by the protozoan parasite *Toxoplasma gondii*, which infects approximately one-third of humans [23]. Usually, toxoplasmosis in immunocompetent people is asymptomatic, whereas it seriously affects immunocompromised individuals, such as AIDS patients, cancer patients, and organ transplant recipients, causing serious medical issues [30]. In particular, this disease may lead to foetal mortality in cases of vertical passage [1]. Unfortunately, current therapeutic options for toxoplasmosis are limited. Antifolate drugs such as pyrimethamine are effective against the tachyzoite stage of *T. gondii* but do not affect the bradyzoite stage that causes chronic infection in the host. Lifelong maintenance with a combination of pyrimethamine-sulfadiazine for toxoplasmic encephalitis often leads to side effects, including severe allergic reactions and haematoxicity [10]. Vaccine inoculation is a promising method in therapeutic applications of toxoplasmosis since vaccination is the most effective and potent strategy for controlling infectious diseases and has saved millions of lives [4]. Consequently, finding and validating vaccine candidates against toxoplasmosis is especially urgent.

Over the past few years, protein vaccines have received increasing attention since they can provoke strong humoral responses by eliciting antigen-specific antibodies [36]. Furthermore, the non-invasive and acceptable route of intranasal vaccination is becoming more attractive because it requires lower doses of antigens, while it triggers mucosal immune responses at local and distant mucosa sites, as well as systemic and cellular immune response [27]. Therefore, several recombinant *T. gondii* proteins prepared by other groups such as rhoptry protein 2 (ROP2) [13], ROP18 [31], malate dehydrogenase (MDH) [19], actin depolymerizing factor (ADF) [18], as well as ours such as actin [44], phosphoglycerate mutase 2 (PGAM 2) [41], ROP17 [42], receptor for activated C kinase 1 (RACK1) [39] and protein disulfide isomerase (PDI) [38] have been tested to assess their immunoprotective effects produced by intranasal immunisation. Although the abovementioned recombinant proteins were capable of triggering both cellular and humoral responses, they all conferred partial protective efficacy against toxoplasmosis.

*Toxoplasma gondii* is a member of the phylum Apicomplexa, which replicates rapidly and requires large amounts of purines for the synthesis of their nucleic acids and other vital components. Nevertheless, it is a purine auxotroph that relies on purine salvage from the host [8]. Pyrimidine salvage in *T. gondii* probably occurs through the following steps: cytidine and deoxycytidine are deaminated by cytidine deaminase to uridine and deoxyuridine, respectively, uridine and deoxyuridine are cleaved to uracil by uridine phosphorylase (UPase), and uracil is metabolised to uridine 5′-monophosphate by uracil phosphoribosyltransferase. Thus, uridine 5′-monophosphate is the end product of both *de novo* pyrimidine biosynthesis and pyrimidine salvage in *T. gondii* [14]. UPase participates in the synthesis of purines and plays an important role in the proliferation of *T. gondii*.

Our previous study showed that *T. gondii* UPase is one of the novel candidate antigens identified among soluble tachyzoite antigens using rabbit anti-*T. gondii* serum by two-dimensional gel electrophoresis and proteomics analyses [20]. The recombinant *T. gondii* uridine phosphorylase (r*Tg*UPase) protein was produced in *Escherichia coli* and showed specific antigenicity [45]. In the present study, r*Tg*UPase was used to intranasally immunise BALB/c mice, and the immune protection against *T. gondii* infection was investigated. The results demonstrated that mice immunised with r*Tg*UPase could protect against *T. gondii* infection by eliciting humoral and mucosal as well as cellular immune responses. Additionally, our data showed that r*Tg*UPase may be a novel vaccine candidate against toxoplasmosis.

## Materials and methods

### Mice, ethics statement and parasites

Female BALB/c mice (6-week-old) were purchased from the Laboratory Animal Center, Shanxi Medical University (Shanxi, China). All mice were maintained under specific-pathogen-free (SPF) conditions and provided with rodent feed and water *ad libitum*. Prior to experiments, the mice were acclimatised for one week. The animal protocols were approved by the Ethics Committee on Animal Research of the Shanxi Medical University (Protocol #: SYDL2021016).

Tachyzoites of the virulent RH strain of *T. gondii* used as a challenge for immunised mice were provided by Peking University Health Science Center (Beijing, China) in this study. Tachyzoites of the highly virulent *T. gondii* RH strain (Type I) were maintained using Vero cells in MEM with 5% FBS (Gibco, USA) and 1% penicillin–streptomycin (Gibco, USA). The *T. gondii* tachyzoites were cultivated every 4 days in Vero cells and collected according to published protocols [25, 26].

### Expression and purification of r*Tg*UPase

r*Tg*UPase was expressed in *E. coli* strain BL21 (DE3) and purified *via* affinity chromatography using nickel-nitrilotriacetic acid (Ni-NTA) agarose (QIAGEN, Hilden, Germany) as described previously [45]. Briefly, total RNA was extracted from tachyzoites of the RH strain of *T. gondii.* A pair of specific primers (sense, 5′–CCGGAATTCATGTCGGAACTCAAAGGAA–3′; antisense, 5′–CCGGCTCGAGTT ACGCCGCAGGCTTGATG–3′) were designed according to the open reading frame of the *Tg*UPase gene (GenBank: DQ385446.1), and the RT-PCR product was cloned into the prokaryotic expression pET-30a(+) vector. The recombinant pET-30a(+)-*Tg*UPase plasmid was transferred into *E. coli* DH5α, and positive clones were selected through colony PCR and confirmed by double restriction enzyme digestion and sequencing. The successful pET-30a(+)-*Tg*UPase construct was transformed into *E. coli* BL21 (DE3) and induced with 0.1 mM IPTG at 37 °C for 4 h for expression. The expressed proteins were analysed by SDS-PAGE with Coomassie blue R-250 staining, and the antigenicity of r*Tg*UPase was analysed with human antiserum of *T. gondii* (1:200) using Western blot assays. r*Tg*UPase was purified via Ni^2+^-NTA agarose (QIAGEN) with 200 mM imidazole elution at 4 °C [38]. Before inoculation into mice or stimulation *in vitro*, a ToxinEraser^TM^ Endotoxin Removal Kit was used to remove endotoxin, and a Chromogenic End-point Endotoxin Assay Kit (Chinese Horseshoe Crab Reagent Manufactory, Xiamen, China) was employed to assess the level of endotoxin remaining in r*Tg*UPase. When the level of endotoxin was less than 0.1 EU/mL, r*Tg*UPase was dialysed against PBS, filtered through a 0.2 μm-pore membrane and stored at −70 °C. r*Tg*UPase was quantified by the Bradford method.

Herein, cultured pET-30a(+)-*Tg*UPase-BL21 (DE3) with or without IPTG induction served as induced or uninduced group, respectively. The bacteria from 1 mL induced or uninduced group were collected by centrifugation and boiled in 100 μL 1 × SDS loading buffer for 5 min, then 10 μL was loaded on SDS-PAGE. The bacteria from 3 mL induced group were harvested via centrifugation, and the obtained pellets were resuspended in cold PBS and homogenised *via* sonication on ice. The lysate was then centrifuged to separate the supernatant and cell pellet. The cell pellet was boiled in 100 μL 1 × SDS loading buffer and then 10 μL was loaded on SDS-PAGE. The supernatant was quantified using BCA method and 10 μg protein was loaded on SDS-PAGE.

### r*Tg*UPase immunisation and sample collection

Forty female BALB/c mice were randomly divided into five groups (8 per group) and intranasally immunised with 20 μL of PBS containing 10 μg, 20 μg, 30 μg or 40 μg r*Tg*UPase on Days 0, 14 and 21. The control group was treated with 20 μL of PBS instead. Two weeks after the third inoculation, the mice were anaesthetised with sodium pentobarbital (1.5%, 0.1 mL/20 g weight, intraperitoneal injection), and blood samples from mice in each group were collected by retro-orbital plexus puncture. The sera were separated and stored at −70 °C until analysed for the presence of specific antibodies.

The spleens were collected under aseptic conditions to perform lymphocyte proliferation assays, and the culture supernatants were used for cytokine assays. Prior to sample collection, the mice were deprived of food for 8 h to deplete the intestinal contents. Nasal, intestinal, vaginal and vesical washes were collected according to a previously described method [44, 47]. All the samples were stored at −70 °C for secretory IgA (sIgA) assays.

### Spleen lymphocyte proliferation assay

According to our previously described method [44], spleens were surgically removed from the mice, and single-cell preparations were pelleted on Day 15 after the last immunisation. In brief, 5 × 10^5^ cells per well were cultured in triplicate in 96-well plates containing RPMI-1640 medium with penicillin–streptomycin (1 mM) and 10% FBS. The culture was stimulated with either 10 μg/mL r*Tg*UPase, 5 μg/mL concanavalin A (Con A) as a positive control or medium alone for proliferation. The plates were incubated in 5% CO_2_ at 37 °C for 4 days. Next, 10 μL of CCK-8 reagent (Dojindo Laboratories, Japan) was added to each well, and the plate was incubated for 3 h. The optical density was then determined at 450 nm using an ELISA reader. The spleen cell proliferative responses were quantitated using a stimulation index (SI), which was calculated as the ratio of the average OD_450_ of the stimulated cells to the average OD_450_ of the unstimulated cells. All assays were performed in triplicate.

### Cytokine assays

Cytokines were measured as previously described [40]. Spleen cells were obtained as described above and cultured in triplicate in flat-bottom 24-well microtiter plates. Supernatants from the cultured splenocytes (1.5 × 10^6^) were collected after 24, 72 or 96 h of stimulation with r*Tg*UPase (10 μg/mL) and assayed for interleukin-2 (IL-2) and IL-4 at 24 h, for IL-10 at 72 h, and for interferon-gamma (IFN-γ) at 96 h. IL-2, IL-4, IL-10 and IFN-γ concentrations were determined using a commercial ELISA Kit (PeproTech, USA), according to the manufacturer’s instructions. Cytokine concentrations were determined by reference to standard curves constructed with known amounts of mouse recombinant IL-2, IL-4, IL-10 and IFN-γ. The sensitivity limits of detection of IL-2, IL-4, IL-10 and IFN-γ were 16, 16, 47 and 23 pg/mL, respectively.

### Specific IgG and sIgA detection

Enzyme-linked immunosorbent assays (ELISAs) were performed for the detection of r*Tg*UPase-specific IgG, IgG1 and IgG2a antibodies in serum samples and sIgA in nasal, intestinal, vaginal and vesical washes collected two weeks after the last immunisation according to our previously described method [41]. Briefly, 96-well flat-bottom microtiter plates were coated with 7.5 μg/mL r*Tg*UPase (100 μL/well) in PBS overnight at 4 °C. The plates were washed with PBS containing 0.05% Tween 20 (PBST), blocked for 1 h at 37 °C in PBS containing 5% FBS, and then washed with PBS. Thereafter, the serum samples (1:200 for IgG, 1:50 for IgG1 and IgG2a) and mucosal washes were incubated in different wells (100 μL/well) for 1 h at 37 °C. After washing, the wells were incubated with 100 μL of goat anti-mouse HRP-IgG, HRP-IgG1, HRP-IgG2a or HRP-IgA (Proteintech, China; diluted 1:2000 in PBS) for 1 h at 37 °C. The plates were washed extensively and incubated with 100 μL of substrate solution for 30 min at 37 °C. The optical density was measured at 492 nm (OD_492_) with a microplate reader (Bio-Tek) followed by 50 μL of 2 N H_2_SO_4_ to stop the enzyme reaction. All the samples were run in triplicate.

### Challenge infection

Two groups of 6-week-old female BALB/c mice (20 mice per group) were vaccinated intranasally with 30 μg of r*Tg*UPase suspended in 20 μL of sterile PBS or 20 μL of PBS and boosted with the same dose three times on Days 0, 14 and 21. The r*Tg*UPase immunisation dose and the immune programme were based on the results of the abovementioned experiment. On Day 15 after the last immunisation, 8 mice in each group were challenged orally with 1 × 10^4^ tachyzoites of the RH strain for the tachyzoite-load assay, while 12 mice were challenged with 4 × 10^4^ tachyzoites for lethal infection.

On the 30th day after being challenged, the infected mice were anaesthetised with sodium pentobarbital, and the numbers of tachyzoites in the livers and brains were measured using real-time PCR assays as previously described [38, 39, 41, 44]. For survival analysis, the acute infected mice were monitored thrice daily until 30 days after the tachyzoite challenge. When painful symptoms were observed, a mouse was moved to an isolated cage for further husbandry; if obvious suffering, such as struggling or whining, was observed, the mouse was sacrificed through carbon dioxide (CO_2_) inhalation.

### Statistical analysis

All statistical analyses were performed using SPSS software for Windows version 19.0. The differences in each variable, including antibody responses, lymphoproliferation assays and cytokine production, among all the groups were compared by one-way ANOVA. The tachyzoite burdens and survival times for vaccinated and control mice were compared using the Kaplan–Meier method. Significant differences in comparisons between groups were defined at *p* < 0.05.

## Results

### r*Tg*UPase was expressed and purified successfully

**Figure 1:**
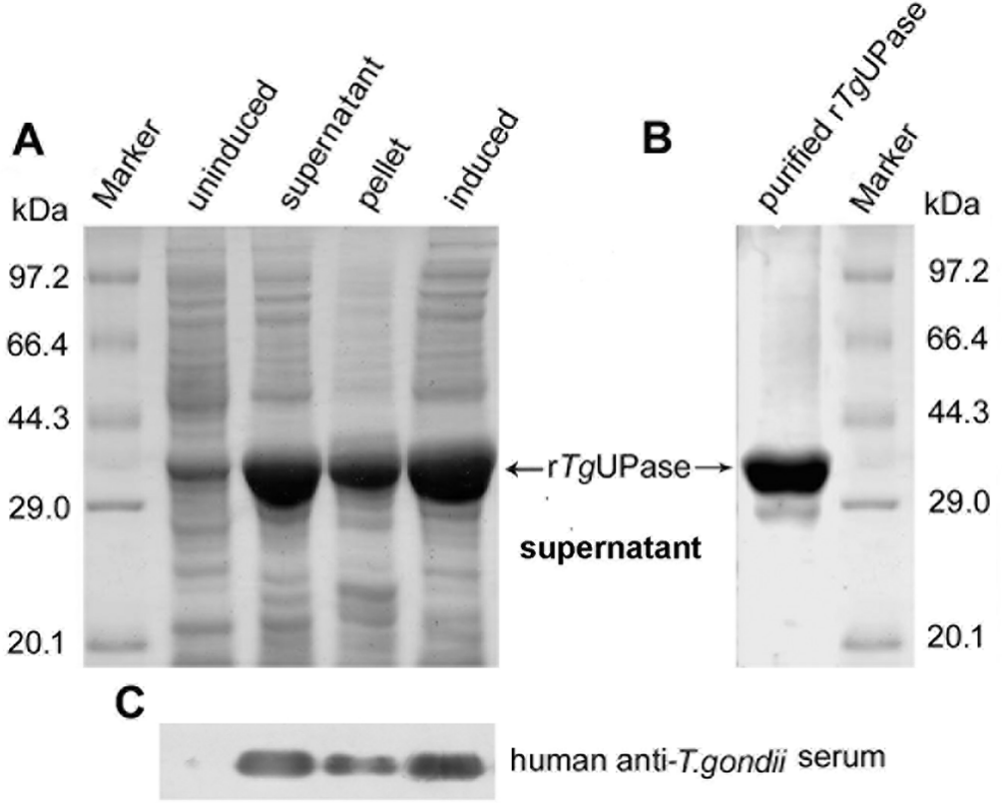
SDS-PAGE and Western blot analyses of the r*Tg*UPase protein. (A) The full-length ORF of *Tg*UPase was expressed in *E. coli*, separated on 12% SDS–PAGE gels and stained with Coomassie blue. The molecular weight of r*Tg*UPase was approximately 38.0 kDa. (B) Purified r*Tg*UPase protein (2 μg/lane) was detected via 12% SDS–PAGE and stained with Coomassie blue, and the purity of r*Tg*UPase was greater than 95%. (C) Western blot analysis of r*Tg*UPase using a human anti-*T. gondii* serum. Left lane: the supernatant (10 μg), Middle lane: cell pellet, right lane: induced whole bacterial protein.

After induction with 0.1 mM IPTG at 37 °C, the r*Tg*UPase proteins were successfully expressed in *E. coli,* and the molecular weight was approximately 38.0 kDa (Fig. 1A). The isolated protein was water soluble and showed greater than 95% purity based on SDS-PAGE analysis (Fig. 1B). Western blot analysis indicated that the r*Tg*UPase band was able to react with anti-*Toxoplasma* human serum (Fig. 1C).

### Systemic immune response induced by r*Tg*UPase vaccination

**Figure 2:**
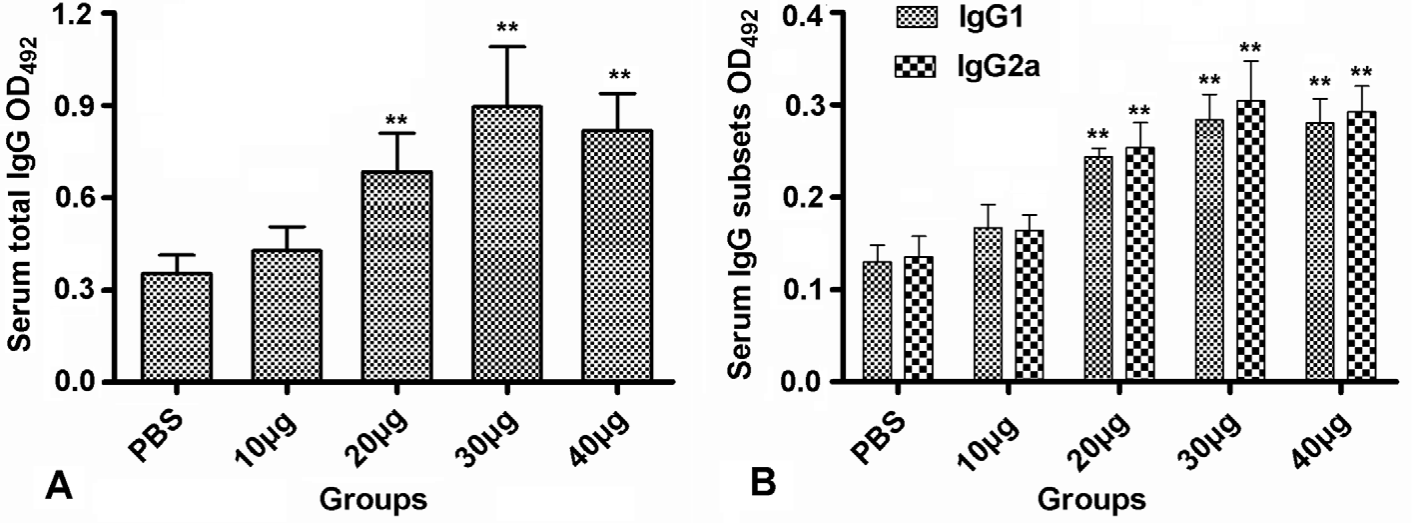
Nasal immunisation induces r*Tg*UPase-specific IgG responses in sera. Titres of both specific total IgG and IgG isotype antibodies in the sera of BALB/c mice were determined by ELISAs with r*Tg*UPase as the bound target two weeks after the last immunisation. (A) Specific total IgG and (B) IgG1 and IgG2a titres in the sera of the mice vaccinated with r*Tg*UPase. The results are expressed as the means of the OD_492_ ± SD (*n* = 8) and are representative of three experiments. **p < 0.01 (vaccinated vs. PBS group).

To assess the systemic immune response in the immunised mice, we evaluated the levels of r*Tg*UPase-specific IgG, IgG1 and IgG2a antibodies in the sera and cytokines from the spleen cell supernatants by ELISAs. The results showed that 20, 30 and 40 μg of r*Tg*UPase could elicit the maximum IgG antibody responses compared to those of the PBS and 10 μg groups (*p* < 0.01), but no significant differences were observed in the IgG responses among the 20, 30 and 40 μg groups and between the PBS and 10 μg groups (*p* > 0.05) (Fig. 2A). A mixed IgG1/IgG2a response was detected in the sera of the mice immunised with r*Tg*UPase (Fig. 2B). Moreover, the mice immunised with 20, 30 and 40 μg r*Tg*UPase elicited higher levels of IgG1 and IgG2a than the controls (*p* < 0.01). The 30 μg group had the highest value but was not significantly different from the 20 and 40 μg groups (*p* > 0.05). The above findings suggested that r*Tg*UPase intranasal immunisation provoked a mixed Th1/Th2 immune response.

### Cellular immune response elicited by r*Tg*UPase inoculation

Spleen cells from 5 groups of mice were prepared 2 weeks after the last immunisation to assess the proliferative responses to r*Tg*UPase. As shown in Table 1, the splenocyte stimulation indices (SI) from the immunised groups were higher than that of the PBS group (*p* < 0.05; *p* < 0.01), and the 30 μg group had the strongest activity vs. all other groups. In addition, splenocytes from each experimental and control group proliferated well in response to ConA (data not shown).

**Table 1:** Lymphocyte proliferation and cytokine production by splenocytes stimulated with r*Tg*UPase.

Groups ^#^	Lymphocyte SI	Cytokine production (pg/mL)^*##*^			

IFN-γ	IL-2	IL-4	IL-10
PBS	0.65 ± 0.67	80.32 ± 78.71	87.33 ± 32.88	123.42 ± 76.5858	74.04 ± 6.34
10 μg r*Tg*UPase	1.45 ± 1.49^a^	130.37 ± 72.78^a^	140.33 ± 65.85^a^	358.57 ± 100.199^b^	79.33 ± 4.66
20 μg r*Tg*UPase	2.44 ± 1.31^b^	206.23 ± 67.20^b^	152.00 ± 28.32^b^	402.70 ± 92.1127^b^	83.67 ± 6.71
30 μg r*Tg*UPase	2.59 ± 1.36^b^	378.14 ± 130.12^b^	221.43 ± 46.91^b^	481.80 ± 64.949 ^b^	86.65 ± 5.52
40 μg r*Tg*UPase	2.35 ± 0.89^b^	354.21 ± 92.57^b^	197.29 ± 24.68^b^	396.24 ± 59.0392^b^	83.64 ± 7.82

# *n* = 8 per group.

## Splenocytes from mice were harvested 2 weeks after the last immunisation. The results are presented as the arithmetic means ± standard errors of three replicate experiments. Values for IFN-γ are for 96 h, values for IL-2 and IL-4 are for 24 h, and values for IL-10 are for 72 h. ^a^: *p <* 0.05 vs. control; ^b^: *p <* 0.01 vs. control.

The cell-mediated immunity produced in the immunised mice was evaluated by measuring the amount of cytokines (IFN-γ, IL-2, IL-4 and IL-10) in the supernatants of stimulated splenocyte cultures from mice of all groups. As shown in Table 1, significantly higher levels of IFN-γ, IL-2 and IL-4 were detected in the mice from all immunised groups compared with the controls (*p* < 0.05; *p* < 0.01). The mice immunised with 30 μg had the highest levels of IFN-γ, IL-2 and IL-4 compared to those of the other dose groups. However, the production of IL-10 did not significantly differ among all groups (*p* > 0.05).

### Mucosal immune responses induced by r*Tg*UPase vaccination

**Figure 3:**
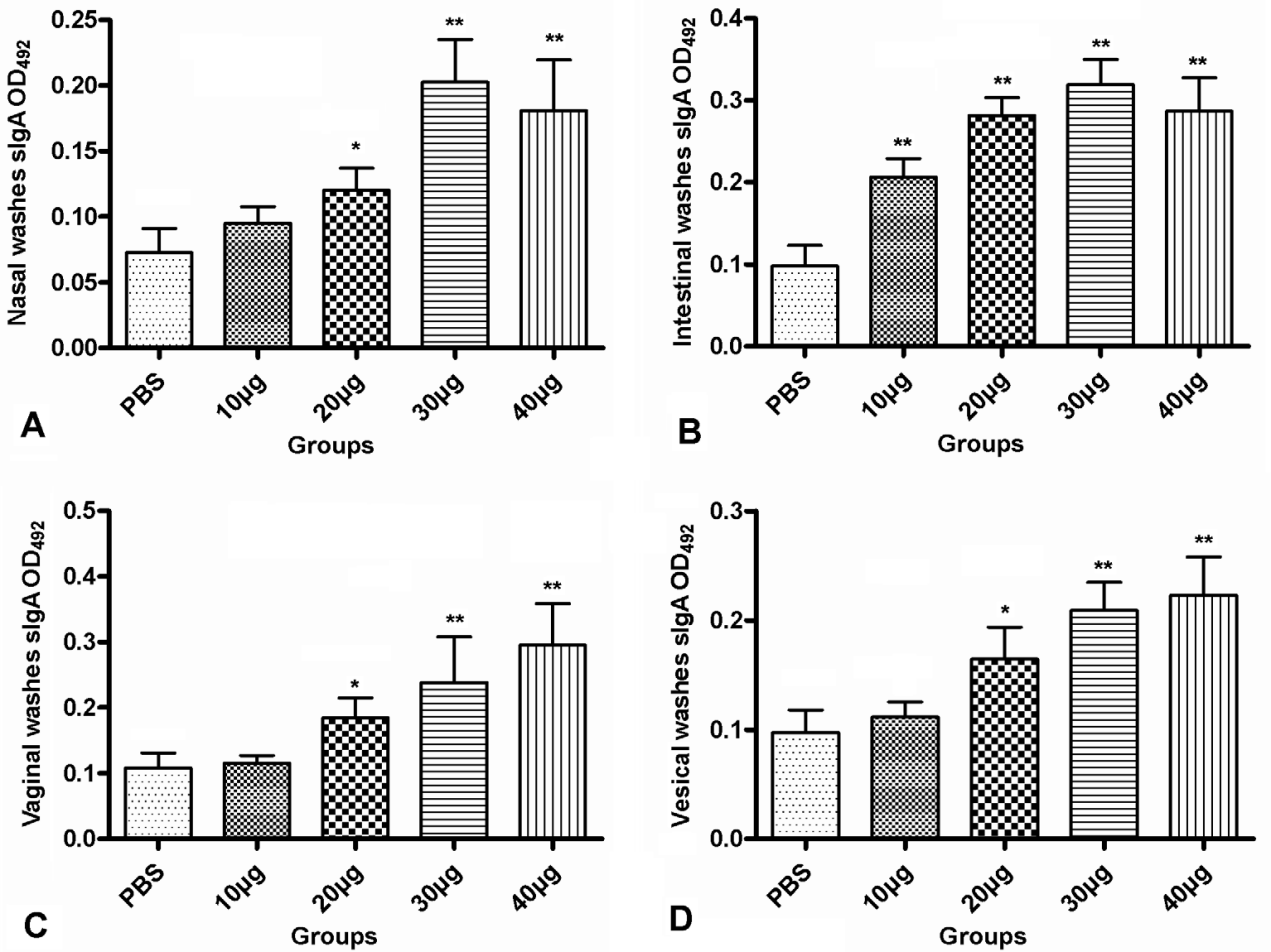
Nasal immunisation induces r*Tg*UPase-specific sIgA responses in mucosal washes. The sIgA antibody titres in mucosal washes from the mice were tested by ELISAs two weeks after the last immunisation. High-level sIgA in (A) nasal washes, (B) intestinal washes, (C) vaginal washes and (D) vesical washes was induced in the mice nasally immunised with r*Tg*UPase compared to those vaccinated with PBS. The data are expressed as the means of the OD_492_ ± SD (*n* = 8) and are representative of three experiments. *p < 0.05, ** *p* < 0.01 (vaccinated vs. PBS group).

To investigate whether the mice immunised with r*Tg*UPase induced mucosal immune responses, we tested the levels of r*Tg*UPase-specific sIgA in the mucosal washes by ELISAs two weeks after the last immunisation (Fig. 3). The titres of r*Tg*UPase-specific sIgA antibody in the mucosal washes were elevated following nasal immunisation. The sIgA antibody titres in the intestinal washes of the 10, 20, 30 or 40 μg r*Tg*UPase-treated groups were significantly higher than those of the PBS control (*p* < 0.01), with the highest titre of sIgA antibody detected in the 30 μg r*Tg*UPase group (Fig. 3B). sIgA levels from the nasal, vaginal and vesical washes were higher in the mice that were nasally immunised with 20, 30 or 40 μg r*Tg*UPase compared with those from the PBS control and 10 μg r*Tg*UPase groups, and 30 μg r*Tg*UPase also provoked the highest sIgA levels in nasal washes (*p* < 0.01), while 40 μg r*Tg*UPase induced the highest sIgA levels in vaginal and vesical washes (*p* < 0.01) (Figs. 3A, 3C and 3D). No significant differences were found between the mucosal washes of the 30 and 40 μg r*Tg*UPase-treated groups. In conclusion, strong mucosal immune responses were elicited by nasal immunisation with r*Tg*UPase at nasal, intestinal, vaginal and vesical mucosal sites.

### Protection against *T. gondii* infection

**Figure 4:**
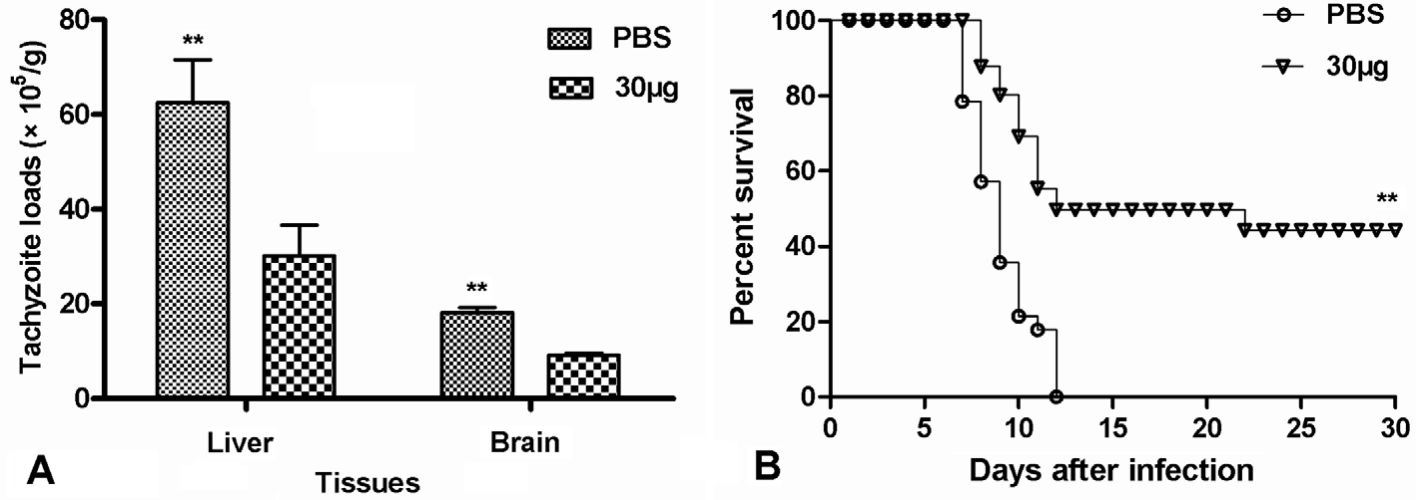
Assay for protection against oral challenge. Mice were nasally immunised with 30 μg r*Tg*UPase or PBS. Two weeks after the last immunisation, mice were orally challenged with tachyzoites. (A) Mice from two groups (*n* = 8 PBS and 8 r*Tg*UPase) were orally infected with 1 × 10^4^ tachyzoites of the *T. gondii* RH strain. Liver and brain tachyzoite burdens were evaluated one month after the challenge. (B) Mouse survival rates of the two groups (*n* = 12 PBS and 12 r*Tg*UPase) were monitored daily after challenge with 4 × 10^4^ tachyzoites of the *T. gondii* RH strain until Day 30 post-challenge. Differences in survival were significant (*p* < 0.01). These results are representative of two independent experiments. Values are the means ± SDs. ** *p* < 0.01. PBS, phosphate buffered saline.

To estimate the protective efficacy of r*Tg*UPase intranasal immunisation against *T. gondii* infection, we generated a mouse model of tachyzoite infection *via* the oral route according to published procedures [41]. Thirty days after the challenge, the tachyzoite loads in the brain tissues were 18.18 (± 1.03) × 10^6^/g in the control group and 9.47 (± 0.16) × 10^6^/g in the r*Tg*UPase-vaccinated group. The tachyzoite loads in the liver tissues were 62.52 (± 8.96) × 10^6^/g in the control group and 30.15 (± 6.46) × 10^6^/g in the r*Tg*UPase-vaccinated group. These data indicated that immunisation with 30 μg of r*Tg*UPase markedly reduced the tachyzoite loads compared to those in the control mice, showing approximately 51.78% (*p* < 0.01) and 49.61% (*p* < 0.05) fewer tachyzoites in the liver and brain, respectively (Fig. 4A).

Additionally, the survival rates of the mice were recorded daily following oral challenge (4 × 10^4^ tachyzoites of the RH strain) until 30 days post-challenge. A significant increase in the survival rate was observed in the 30 μg r*Tg*UPase-immunised group compared to the control group (*P* < 0.01) (Fig. 4B). The mice immunised with 30 μg of r*Tg*UPase had a significantly increased survival rate (44.29%) on the 30th day after challenge, and all mice in the PBS group died within 12 days post-challenge. These results demonstrated the protective effect of r*Tg*UPase against *T. gondii* RH strain challenge.

## Discussion

*Toxoplasma gondii* is an obligate intracellular protozoan that infects almost all warm-blooded animals, including humans. There are three asexual stages, including sporozoite, bradyzoite and tachyzoite, that can invade the cells in the *T. gondii* life cycle [22]. During these asexual stages, sporozoites are produced by sexual reproduction and excreted in the oocysts through felid faeces; bradyzoites are a slow multiplication form coming from the tissue cysts when chronic infection occurs; and tachyzoites, a proliferative form that causes a decrease in cholesterol content in the liver and brain and a decrease in host immune functions, leading to acute infection [37]. Importantly, tachyzoites can be maintained and produced *in vitro* (cellular cultivation systems) and *in vivo* (animal models), which makes them the most experimentally manipulable form that has been extensively modelled in many studies, including antigen production, immunological studies, drug trials *in vitro* and others [3]. Given these aspects of the tachyzoite, in the present study, *T. gondii*-infected mouse models were employed using tachyzoites. Of course, another consideration is that the antigen r*Tg*UPase, which converts uridine or deoxyuridine to uracil and participates in the pyrimidine salvage pathways of *T. gondii*, originates from tachyzoites [11, 20].

*Tg*UPase has been identified from soluble tachyzoite antigens and can react with a rabbit anti-*T. gondii* serum [20]. Moreover, r*Tg*UPase could react with human anti-*T. gondii* serum [45], indicating that this protein could be used as a novel vaccine candidate. Here, r*Tg*UPase nasal inoculation in BALB/c mice enhanced the output of sIgA in mucosal tracks and the production of total IgG and IgG isotypes (IgG1 and IgG2a) in sera. Additionally, r*Tg*UPase stimulated lymphocyte proliferation and the release of the cytokines IFN-γ, IL-2, and IL-4 but not IL-10 in isolated spleen cells from inoculated mice. Decreased loads of tachyzoites in host tissues, an enhanced host survival rate (44.29%) and prolonged survival time were observed in the r*Tg*UPase-vaccinated mice challenged with *T. gondii* infection. These data demonstrate that r*Tg*UPase is a promising immunogen for developing a mucosal vaccine against *T. gondii* infection.

The natural portal of infection of *T. gondii* is the oral route by which either cyst-contaminated meats or oocyst-polluted fruits and vegetables are ingested [43]. In previous papers, tachyzoites have been proven to infect cats and mice via the oral route [9, 29, 41]. After ingestion, *T. gondii* targets the small intestine for infection. Given the mucosal exposure to *Toxoplasma*, mucosal immunity is believed to protect against *Toxoplasma* infection [46]. IgA is the most abundant antibody isotype in mucosal immunity to many pathogens, including *Toxoplasma,* which invades the host organism by crossing mucous membranes [12]. sIgA, which is the major effective form of mucosal IgA, annihilates pathogens with immune exclusion via nonspecific immunity [6]. Additionally, sIgA plays an indispensable role in specific immunity provoked by pathogens or mucosal vaccines [16]. At the site of entry, sIgA, which is synthesised from the epithelial cells of the intestine, can partially eliminate *T. gondii* via nonspecific immune exclusion and/or a specific neutralising role [16]. Our present results revealed that sIgA antibodies in intestinal washes as well as in nasal, vaginal and vesical washes were significantly enhanced in 30 μg r*Tg*UPase-vaccinated mice. These data indicate that local mucosal inoculation with recombinant *T. gondii* protein can elicit *in situ* and distal immune responses. Therefore, mucosal inoculation via the intranasal route has extensive potential for sparking a protective immune response in all mucosal compartments.

Apart from sIgA, IgG, another element in humoral immunity, also participates in resistance to *T. gondii* [35]. The isotypes IgG, IgG1 and IgG2a can play an important role in resistance to *T. gondii* through complement fixation, opsonisation, or antibody (Ab)-dependent cell cytotoxicity [7]. Here, immunisation of mice with 30 or 40 μg of r*Tg*UPase led to the development of higher levels of total r*Tg*UPase-specific IgG antibodies compared with those of the PBS and 10 and 20 μg groups. Furthermore, a mixed humoral response of both IgG1 and IgG2a was unveiled in the 30 or 40 μg r*Tg*UPase-vaccinated mice, indicating that r*Tg*UPase immunisation predominantly activated a mixed Th1/Th2 immune response.

Cell-mediated immunity plays a major role in anti-*T. gondii* immunity. Among the cytokines produced by activated immune cells such as natural killer (NK) cells and CD4^+^ and CD8^+^ T lymphocytes, IFN-γ plays a pivotal role in the host defence against *T. gondii* infection [33]. IFN-γ controls tachyzoite replication during both acute and chronic phases of infection and prevents reactivation of *T. gondii* from dormant cysts at a later phase [2]. IFN-γ also promotes robust production of indoleamine 2,3-dioxygenase (IDO), which suppresses *T. gondii* growth [24]. In addition, IFN-γ-stimuslated cells express copious IFN-stimulated proteins, including GTPase family members, such as immunity-related GTPases (IRGs), which accumulate on the *T. gondii* PV membrane (PVM), destroy this structure, and then kill the parasites [32]. IL-2 can drive NK and CD8^+^ cell expansion, which is responsible for controlling tachyzoite proliferation via the synthesis of IFN-γ [34]. IFN-γ and IL-2 can stimulate the induction of antigen-specific sIgA responses that control the invasion of *T. gondii* at intestinal mucosal sites [39]. IL-4 plays a major role in controlling the development of cell-mediated immunity (CMI) and is involved in protection against the development of toxoplasmic encephalitis by preventing the formation of *T. gondii* cysts and the proliferation of tachyzoites in the brain [5]. IL-10 is a major antagonist involved in modulating IFN-γ, avoiding an extreme immune response that causes extensive inflammation and host tissue damage [28]. In our study, the production of IFN-γ, IL-2 and IL-4 rather than IL-10 in the supernatant of cultured spleen cells was significantly increased in the r*Tg*UPase-vaccinated mice, suggesting that Th1- and Th2-type cellular-mediated immune responses were generated. Furthermore, the elevated number of spleen lymphocytes was consistent with the augmentation of cytokines in different groups stimulated with r*Tg*UPase. These cytokines are valid evaluation indicators for the actual response in an *in vivo* assay to assess the immune responses of novel vaccine candidates against toxoplasmosis [5]. The above findings suggested that r*Tg*UPase intranasal inoculation could elicit cell-mediated immune responses.

Furthermore, we prepared *T. gondii* RH strain infection mouse models *via* peroral infection of tachyzoites to assess the protective effect of the r*Tg*UPase protein against *T. gondii* infection. Obvious reductions in tachyzoites were observed in the liver and brain tissues of the r*Tg*UPase-vaccinated mice compared with those in the control mice. Additionally, the immunised mice exhibited significant protection against lethal tachyzoite infection, showing an approximately 44.29% improved survival rate and prolonged lifespan. In conclusion, *Tg*UPase immunisation is effective in decreasing the loads of tachyzoite infection in host tissues and partially protects the host against *T. gondii* infection. Apart from type I strains, type II strains have low virulence, and type III strains are avirulent [11]. For further evaluation of the protective effect of r*Tg*UPase, a type II or type III strain of *T. gondii* should be used to challenge mice in the next study.

Our present and previous results as well as others’ data demonstrated that intranasal inoculation with recombinant *T. gondii* proteins all elicited antigen-specific IgG and sIgA antibodies, cellular cytokines with a Th1-oriented immune responses. However, the immune protective efficacy was different. r*Tg*UPase showed a 44.29% improved survival rate, greater than r*Tg*PDI (31%) [38], r*Tg* ADF (36.36%) [18], roughly the same as r*Tg*RACK1 (45%) [39], r*Tg*MDH (47%) [19], but less than r*Tg*ACT (50%) [44], rTgROP17 (59.17%) [42] and r*Tg* PGAM 2 (70%) [41]. Compared with a cocktail vaccine composed of multicomponent proteins such as *Tg*MIF, *Tg*CDPK3, and *Tg*14-3-3 which provoked higher serum antibody titers and higher survival rate (90%) [17], a single recombinant protein vaccine in the present study and in our previous studies elicited lower serum antibody titers and lower survival rate. Henceforth, the study of multicomponent cocktail vaccines will be the focus on this issue.

To date, a *T. gondii* vaccine for clinical application is not yet available, although multiple vaccine candidates have been suggested. The reason is the existence of both multiple antigenically distinct strain types and multifarious antigenically diverse developmental stages. Moreover, *Toxoplasma* can effectively escape the immune system by developing a chronic encysted stage that is not easily eliminated by immune responses. An effective vaccine can prevent the tachyzoites from gaining access to other host tissues and to the placenta. Additionally, this vaccine can cause an immune response able to stop and kill the parasite when it penetrates the intestinal barrier. Of course, an effective vaccine must induce an immune response able to inhibit the formation of tissue cysts during the chronic phase of toxoplasmosis. For these demands, multicomponent antigens from oocysts, bradyzoites, tachyzoites and cysts should be considered. In addition, appropriate adjuvants such as CpG-containing oligodeoxynucleotide (CpG ODN) [15] and nanocurcumin [21] should be taken into account.

Taken together, our findings indicate that intranasal inoculation with r*Tg*UPase provokes both mucosal and systemic as well as cellular-mediated immune responses, which confer protection against *T. gondii* RH strain infection in BALB/c mice. Although the protective effect is partial, findings from the present study indicate that r*Tg*UPase is a promising vaccine candidate against *Toxoplasma*.

## Conflict of interest

The authors declare that they have no competing interests.
